# Integrated Process for Capture and Purification of Virus-Like Particles: Enhancing Process Performance by Cross-Flow Filtration

**DOI:** 10.3389/fbioe.2020.00489

**Published:** 2020-05-25

**Authors:** Nils Hillebrandt, Philipp Vormittag, Nicolai Bluthardt, Annabelle Dietrich, Jürgen Hubbuch

**Affiliations:** Institute of Engineering in Life Sciences, Section IV: Biomolecular Separation Engineering, Karlsruhe Institute of Technology (KIT), Karlsruhe, Germany

**Keywords:** virus-like particles, precipitation, cross-flow filtration, integrated processing, downstream processing

## Abstract

Virus-like particles (VLPs) are emerging nanoscale protein assemblies applied as prophylactic vaccines and in development as therapeutic vaccines or cargo delivery systems. Downstream processing (DSP) of VLPs comes both with challenges and opportunities, depending on the complexity and size of the structures. Filtration, precipitation/re-dissolution and size-exclusion chromatography (SEC) are potent technologies exploiting the size difference between product and impurities. In this study, we therefore investigated the integration of these technologies within a single unit operation, resulting in three different processes, one of which integrates all three technologies. VLPs, contained in clarified lysate from *Escherichia coli*, were precipitated by ammonium sulfate, washed, and re-dissolved in a commercial cross-flow filtration (CFF) unit. Processes were analyzed for yield, purity, as well as productivity and were found to be largely superior to a reference centrifugation process. Productivity was increased 2.6-fold by transfer of the wash and re-dissolution process to the CFF unit. Installation of a multimodal SEC column in the permeate line increased purity to 96% while maintaining a high productivity and high yield of 86%. In addition to these advantages, CFF-based capture and purification allows for scalable and disposable DSP. In summary, the developed set-up resulted in high yields and purities, bearing the potential to be applied as an integrated process step for capture and purification of *in vivo*-assembled VLPs and other protein nanoparticles.

## Introduction

Vaccination has reduced morbidity and mortality world-wide, especially since the introduction of the World Health Organization’s Expanded Program on Immunization (Greenwood, 2014). Expansion of the vaccine portfolio by virus-like particles (VLP) has opened up new opportunities, such as the prevention or treatment of cancer (Ding et al., 2009; Klamp et al., 2011; Goldinger et al., 2012; Bryan et al., 2016; Lizotte et al., 2016; Bolli et al., 2018; Palladini et al., 2018; Mohsen et al., 2019a, b). However, especially VLP downstream processing (DSP) faces major challenges, such as low yields and the lack of platform processes or rapid analytical techniques. This is due to the complexity of the product and the associated processes, resulting in high development and production costs (Ladd Effio and Hubbuch, 2015). The structural properties of VLPs are similar or identical to the corresponding virus structure they are derived from Zeltins (2013). Composed of at least one type of viral structural protein, they are in a size range of approximately 25 to 200 nm (Chung et al., 2010; Reiter et al., 2019). Incorporation of foreign epitopes into VLP-forming viral structural proteins results in so-called chimeric VLPs (Pumpens and Grens, 2001). In a previous study, we observed that upon insertion of smaller peptides, the size of chimeric Hepatitis B core antigen (HBcAg) VLPs remained comparable to native HBcAg VLPs with a diameter of 31 ± 2 to 33 ± 3 nm (Selzer and Zlotnick, 2017; Rüdt et al., 2019). During production, the size difference between VLPs and host cell proteins (HCPs) as well as other smaller contaminants can be exploited for DSP of VLPs (Ladd Effio and Hubbuch, 2015).

**FIGURE 1:**
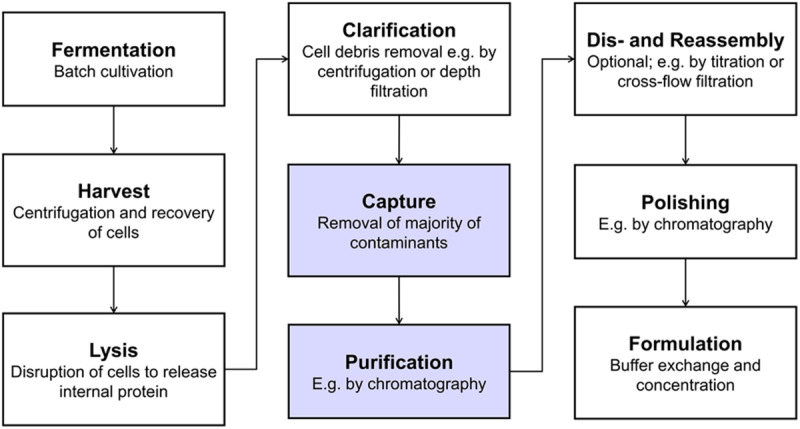
Typical production process for intracellularly produced, *in vivo*-assembled virus-like particles (VLPs). Virus structural proteins can be expressed in a variety of host systems, such as *E. coli*, yeast or plant cells (Ladd Effio and Hubbuch, 2015). After harvest and lysis, cell debris are removed by solid-liquid separation and the VLPs remain in solution. VLPs are then captured and purified, followed by an optional dis- and reassembly step, which has shown to increase VLP stability, homogeneity and immunogenicity (Mach et al., 2006; Klamp et al., 2011; Zhao et al., 2012). Finally, the product is polished and formulated. The process steps that were investigated as integrated unit operations in this study are highlighted in blue.

A typical VLP production process is shown in Figure 1 including unit operations such as centrifugation, filtration, and chromatography. Bind and elute chromatography, the work horse in biopharmaceutical manufacturing for capture, purification, and polishing, suffers from low dynamic binding capacities (Ladd Effio and Hubbuch, 2015), diffusion limitations (Kramberger et al., 2015), and often too small pore sizes (Kattur Venkatachalam et al., 2014) for the purification of VLPs. Size differences between VLPs and the bulk of host cell contaminants can be exploited by size-sensitive techniques such as size-exclusion chromatography (SEC) – especially for analytical purposes (Ladd Effio et al., 2016) – precipitation, filtration, and ultracentrifugation (Ladd Effio and Hubbuch, 2015). While ultracentrifugation is applied to lab-scale processes (Jiang et al., 1992; Mason et al., 1996; Ausar et al., 2006), scalability and variability issues, among others, hamper its application to industrial-scale processes (Koho et al., 2012; Kleiner et al., 2015).

Originally developed for the fractionation of blood by Edward Cohn and coworkers in the 1940s (Cohn, 1941; Cohn et al., 1946), precipitation of contaminants or native precipitation of the product are promising alternatives for protein separation and purification (Martinez et al., 2019). In this context, native precipitation has been reported as highly selective for VLPs (Tsoka et al., 2000; Kim et al., 2010; Koho et al., 2012; Zahin et al., 2016), since larger proteins or protein assemblies are more susceptible to precipitation (Rothstein, 1993). The steric exclusion effect associated with the frequently applied precipitant polyethylene glycol (PEG) generally leads to steeper slopes in the precipitation curves for larger proteins (Iverius and Laurent, 1967; Sim et al., 2012). For precipitation with kosmotropic salts, surface charge is, however, thought to have a greater effect than size (Curtis et al., 1998). Separation of product-containing precipitate and supernatant can be achieved by centrifugation or filtration. While PEG has been successfully applied to VLP precipitation (Tsoka et al., 2000; Koho et al., 2012), its application is limited when filtration is used as solid-liquid separation technique, as filtration performance is impaired by a PEG-induced viscosity increase (Plisko et al., 2016; Li and Zydney, 2017). Next to PEG of various molecular weights, the kosmotropic salt ammonium sulfate [(NH_4_)_2_SO_4_] is a commonly applied precipitant (Kim et al., 2010; Zahin et al., 2016; Kazaks et al., 2017). In a study on adenovirus (Schagen et al., 2000), dead-end filtration has been applied to retain (NH_4_)_2_SO_4_-precipitated virus but exhibited only 46-61% recovery from the filter. As an alternative to dead-end filtration, cross-flow filtration (CFF) in diafiltration (DF) mode has been applied to recover precipitated monoclonal antibodies (mAbs) (Venkiteshwaran et al., 2008; Kuczewski et al., 2011; Hammerschmidt et al., 2016). Precipitate was retained by a microfilter, allowing for a wash in DF mode. In CFF, turbulent flow along the membrane surface ensures better recovery from the filter (Davies and Smith, 2010), also reducing concentration polarization and fouling (van Reis and Zydney, 2007). A main advantage of precipitate recovery by CFF over centrifugation lies in avoiding the compaction of precipitate that occurs during centrifugation, which allows for shorter precipitate re-dissolution times using CFF (Hammerschmidt et al., 2016). Additionally, in the above-mentioned studies, precipitation and wash were conducted as integrated CFF-based process steps that showed a higher wash efficiency as compared to centrifugation (Kuczewski et al., 2011; Hammerschmidt et al., 2016). In these studies, the precipitate was re-dissolved by dilution.

This said, it seems promising to dissolve precipitated product by DF into a re-dissolution buffer. Product could subsequently be recovered in the permeate stream as it passes the microfilter. Implementing this approach, the permeate can be separated into fractions allowing for purity increase and concentration adjustment by strategic pooling while undissolved contaminants are retained by the microfilter.

In our experience with DSP of *Escherichia coli* (*E. coli*)-derived VLPs, HCP reduction poses a minor challenge as compared to nucleic acid depletion, demanding for a purification method to reduce the nucleic acid burden. One commonly applied strategy is the supplementation of lysate with Benzonase, a nucleic acid digestion enzyme. In recent years, a novel multimodal SEC (mmSEC) medium Capto Core 400/700 has been developed that found successful application in the purification of VLPs, decreasing impurity levels significantly (Zhao et al., 2015; Lagoutte et al., 2016; Somasundaram et al., 2016). Integration of a precipitation, wash, and re-dissolution step on a CFF system together with this novel mmSEC medium seems therefore promising.

**FIGURE 2:**
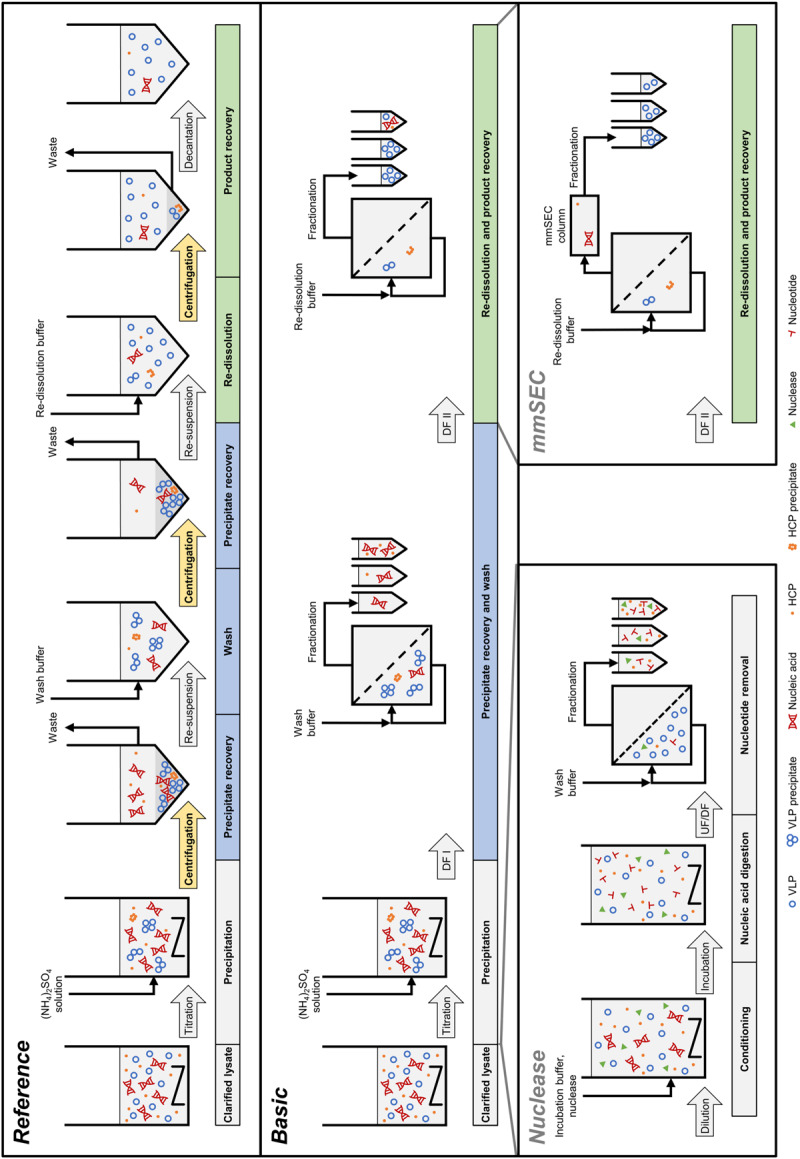
Schematic overview of the processes investigated in this study. The *Reference* process is shown at the top, consisting of centrifugation-based precipitation, wash, and re-dissolution. Process transfer to a cross-flow filtration (CFF) unit resulted in the *Basic* process. Transferred process steps are wash and re-dissolution, highlighted in blue and green, respectively. Wash and re-dissolution are multiple process steps consisting of repeated centrifugation (highlighted in yellow) in the *Reference* process. In the *Basic* process, these are reduced to two consecutive diafiltration (DF) steps by simply switching between diafiltration buffers (Figure 3). Alternative CFF process variants, either *Nuclease* or *mmSEC*, are modifications from the *Basic* process. The *Nuclease* process adds a nucleic acid digestion and a 300 kDa wash step preceding precipitation and continues like the *Basic* process. The *mmSEC* process sequence is identical to the *Basic* process sequence but has a modified re-dissolution step (DF II) including a multimodal size-exclusion chromatography (mmSEC) column in the permeate line. (NH_4_)_2_SO_4_, ammonium sulfate; HCP, host cell protein; UF/DF, ultrafiltration/diafiltration; VLP, virus-like particle.

In the light of the above, the objective of our study was to develop an integrated membrane-aided precipitation, wash, and re-dissolution process for capture and purification of VLPs. The set-up was realized on a commercial CFF unit coupled to a basic preparative chromatography system for monitoring of ultraviolet (UV) absorbance at 280 nm and fractionation. Three process variants were developed, the simplest of which comprised precipitation, wash, and re-dissolution within an integrated CFF-based set-up (Figure 2, Process *Basic*). To improve product purity, this method was further either extended by installation of a Capto Core 400 column in the CFF permeate line (Process *mmSEC*) or by pretreatment of the lysate with Benzonase prior to the precipitation step (Process *Nuclease*). As a model VLP, a C-terminally truncated chimeric HBcAg VLP was investigated. The three process variants were compared to a centrifugation-based precipitation, wash and re-dissolution process (Process *Reference*).

## Materials and Methods

### Materials, Buffers, and VLPs

All chemicals were purchased from Merck Millipore (Darmstadt, Germany), unless otherwise stated. Solutions and buffers were prepared with ultrapure water (PURELAB Ultra, ELGA LabWater, Lane End, United Kingdom). A buffer consisting of 50 mM Tris, 100 mM NaCl, 1 mM EDTA (AppliChem GmbH, Darmstadt, Germany), pH 8 was used as lysis buffer. The wash buffer was created from lysis buffer that was adjusted to 0.25% (v/v) polysorbate 20 (AppliChem GmbH, Darmstadt, Germany) with a 10% (v/v) polysorbate 20 stock solution and to 150 mM (NH_4_)_2_SO_4_ (AppliChem GmbH, Darmstadt, Germany) with a 1 M (NH_4_)_2_SO_4_ stock solution. In the *Nuclease* process and respective experiments, the digestion and nuclease wash buffers were both 50 mM Tris at pH 8, containing 20 mM NaCl, 0.2 mM EDTA, and 2 mM MgCl_2_. The re-dissolution buffer was 50 mM Tris at pH 8 for all experiments. All buffers were pH-adjusted with 32% HCl. BioNTech Protein Therapeutics generously provided the chimeric HBcAg VLP plasmid. HBcAg was expressed in *E. coli* and liberated by lysis as described in Supplementary Information S1. Its extinction coefficient at 280 nm of 1.558 L g^–1^ cm^–1^ was derived from the web-tool ProtParam (Gasteiger et al., 2005) and used for all methods. *E. coli* lysate was diluted to ensure a consistent HBcAg content, resulting in HBcAg concentrations between 2.60 and 2.66 g/L, used as lysate for all processes and experiments.

### Precipitation and Re-dissolution Screening

For processes *Reference*, *Basic*, *mmSEC*, and *Nuclease*, optimal parameters for the precipitation were determined in screening experiments. Screening experiments for precipitant concentration were performed at a small scale in reaction tubes. Lysate was used either untreated or pretreated. Pretreatment comprised overnight dialysis with Slide-A-Lyzer G2 cassettes (10 kDa, 3 mL, Thermo Scientific, Rockford, IL, United States) into the digestion buffer with or without addition of >114 U/mL of Benzonase (Sigma Aldrich, Saint Louis, MO, United States) to the lysate. In 1.5 mL reaction tubes, 170 or 200 μL of these solutions, adjusted to 0.25% (v/v) polysorbate 20, were mixed with different volumes of (NH_4_)_2_SO_4_ stock solution and incubated for 30 min at room temperature (RT), which was between 22 and 23°C for all experiments. The solution was spun down at 17000 rcf for 2 min in a tabletop centrifuge Heraeus Pico 17 (Thermo Electron LED GmbH, Osterode am Harz, Germany) and the supernatant was recovered. For screening of the incubation time during precipitation, untreated lysate was precipitated in a 20 mL batch, sampled at 10 min intervals, and treated as described above.

Small-scale re-dissolution experiments were conducted to test the influence of solution components on re-dissolution efficiency. Pooled fractions F3-F11 of the *mmSEC* process were concentrated to 7.74 g/L using 20 mL VivaSpins with 100 kDa MWCO (Sartorius Stedim Biotech GmbH, Göttingen, Germany). In 1.5 mL tubes, 0.5 mL of concentrated HBcAg solution was mixed with 0.5 mL of five different solutions. Solutions were (a) 200 mM NaCl, 50 mM Tris, 2 mM EDTA, pH 8.0, (b) 40 mM NaCl, 50 mM Tris, 2 mM EDTA, pH 8.0, (c) 200 mM NaCl, 50 mM Tris, 0.4 mM EDTA, 4 mM MgCl_2_, pH 8.0, (d) supernatant of the precipitation step during the *Reference* (section “Centrifugation-Based Wash and Re-dissolution”) process, and (e) supernatant of the wash step during the *Reference* process. Solutions were adjusted to 0.25% (v/v) polysorbate 20 and then to 150 mM (NH_4_)_2_SO_4_ for precipitation. Samples were incubated for 30 min at 300 rpm and 23°C in a thermo-shaker Thermomixer comfort (Eppendorf, Hamburg, Germany) and subsequently centrifuged at 15294 rcf in an Eppendorf 5810R centrifuge for 20 min at 20°C. Supernatant was removed by pipetting. A volume of 1 mL re-dissolution buffer was added and the pellet was resuspended. The reaction tubes were incubated at 10 rpm at RT in an overhead shaker LD-79 (Labinco, Breda, Netherlands) for 60 min, centrifuged with identical settings, and the supernatant was recovered.

### CFF Instrumentation and Set-Up

**FIGURE 3:**
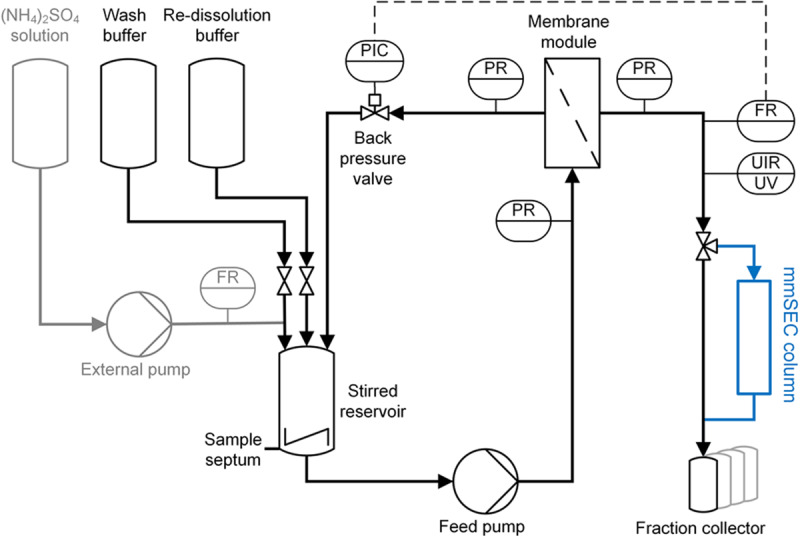
Piping and instrumentation diagram (P&ID) of the precipitation and cross-flow filtration (CFF) setup. The set-up used for wash and re-dissolution of the CFF processes *Basic* and *Nuclease* is shown. For process *Nuclease*, the depicted set-up was used with different membranes (300 kDa and 0.2 μm) for the respective wash steps. The *mmSEC* process included an additional multimodal size-exclusion chromatography column (mmSEC) in the permeate stream, highlighted in blue. The precipitation set-up consists of the components highlighted in gray on the left and the stirred reservoir. Precipitant was ammonium sulfate [(NH_4_)_2_SO_4_]. Gray highlighted components were removed after completion of precipitation. C, control; F, flow rate; I, indicate; P, pressure; R, record; U, multivariable; UV, ultraviolet.

The CFF precipitation, wash, and re-dissolution set-up (Figure 3) was based on a KrosFlo Research KRIIi CFF system with an automatic backpressure valve (Spectrum Labs, Rancho-Dominguez, CA, United States), a stirred cell (Sartorius Stedim Biotech GmbH, Göttingen, Germany) as reservoir, and 0.2 μm 200 cm^2^ Hydrosart or 300 kDa MWCO 200 cm^2^ polyether sulfone (PESU) membranes (both Sartocon Slice 200) with corresponding membrane holders (all Sartorius Stedim Biotech GmbH, Göttingen, Germany). The three stirred cell inlet ports were connected to retentate, wash buffer, and re-dissolution buffer lines. A Sensirion Liquid Flow Meter SLS-1500 (Sensirion AG, Stäfa, Switzerland) was installed at the permeate outlet of the membrane holder and connected with a 1/16” PEEK capillary with 0.75 mm inner diameter to the wash valve of an ÄKTA Start (GE Healthcare, Uppsala, Sweden). On-line ÄKTA Start UV sensor data were converted to on-line concentration data applying Beer’s law using the HBcAg extinction coefficient. The permeate was fractionated in either 15 mL (wash) or 5 mL (re-dissolution) fractions in 15 mL tubes (Corning, Reynosa, TAM, Mexico). In all presented filtration processes, a constant permeate flow rate of 2 mL/min was set and maintained using the automatic backpressure valve either by manual valve control (Process *Basic*) or automatic control (Processes *mmSEC* and *Nuclease*). Therefore, the backpressure valve controller was fed with flow rate data of the flow meter (at >1 Hz) instead of transmembrane pressure data as in normal operation mode using a custom-written communication MATLAB 2018b script (The Mathworks, Natick, MA, United States). Flow rate, path, and control were optimized in pre-experiments, and data were temporally aligned considering delay volumes (for more detail see Supplementary Information S2).

### Precipitation, Wash, and Re-dissolution Process by CFF

Diluted lysate, adjusted to 0.25% (v/v) polysorbate 20, was filled into the aforementioned stirred cell with three inlets and two outlets. One outlet was capped with an injection plug (Fresenius Kabi, Bad Homburg, Germany) for sampling, the other outlet either closed or connected to the suction port of the CFF feed pump. A Minipuls 3 peristaltic pump (Gilson, Villiers le Bel, France) was used to pump 1 M (NH_4_)_2_SO_4_ solution at 1 mL/min through one of the inlet ports of the cell up to a final concentration of 150 mM (NH_4_)_2_SO_4_ (Figure 3). The flow rate was monitored using a Sensirion Liquid Flow Meter SLS-1500. The stirred cell was set to minimal stirring speed. The solution was incubated for 30 min at RT. During incubation, 250 μL samples were taken every 10 min.

Three wash and re-dissolution process variants were examined, referred to as *Basic*, *mmSEC*, and *Nuclease* (Figure 2). The *Basic* process consisted of wash and re-dissolution of precipitate suspension by constant volume DF against wash and re-dissolution buffer, respectively, and fractionation of the permeate. CFF feed flow rate in all filtration steps was 30 mL/min. Compared to the *Basic* process, the *mmSEC* process included a Capto Core 400 HiScreen column (GE Healthcare, Uppsala, Sweden) with a nominal column volume of 4.7 mL in the permeate line downstream of the fractionation valve of the ÄKTA Start (Figure 3). The *Nuclease* process was conducted like the *Basic* process with additional pretreatment of the lysate prior to precipitation. The lysate was diluted 1:5 with a buffer containing 50 mM Tris and 2.5 mM MgCl_2_ at pH 8 to optimize the conditions for the digestion of nucleic acids by Benzonase, resulting in the composition of the digestion buffer. Benzonase was added to a concentration of ≥114 Units/mL and incubated overnight for 16 h at 80 rpm and 23°C in a 225 mL tube in a MaxQ 6000 Shaker (Thermo Scientific, Marietta, OH, United States). The solution was concentrated five-fold by ultrafiltration (UF) in the CFF unit with the 300 kDa membrane. The solution was diafiltered for five diafiltration volumes using nuclease wash buffer. The permeate of UF and DF was fractionated into 15 mL fractions. The retentate was processed analogous to the lysate in the other processes.

### Centrifugation-Based Wash and Re-dissolution

In a centrifugation-based process (Figure 2, process *Reference*), precipitation was performed identically to the experimental procedure for the CFF runs, whereas wash and re-dissolution were performed as a centrifugation protocol. The suspension of 20 mL was centrifuged at 17387 rcf at 20°C for 20 min. Supernatant was removed and the pellet was resuspended. The procedure including centrifugation and resuspension was repeated with re-dissolution buffer. The suspension was transferred into a stirred cell and stirred at minimal speed. After 1, 2, and 3 h, a sample was taken, spun down at 17000 rcf for 2 min in the tabletop centrifuge, and the supernatant was recovered.

### Analytical Characterization

Size-exclusion chromatography (SEC) was coupled with a diode array detector (DAD), multi-angle light scattering (MALS), and quasi-elastic light scattering (QELS) to quantify and specify differently sized species. An Agilent BioSEC-5 4.6 × 300 mm, 5 μm, 1000 Å column (Agilent, Santa Clara, CA, United States) was used at a Dionex Ultimate 3000 RS UHPLC system controlled by Chromeleon version 6.8 SR15 (Thermo Fisher Scientific, Waltham, MA, United States). The method was isocratic for 14 min at a flow rate of 0.4 mL/min with 50 mM potassium phosphate buffer at pH 7.4. The injection volume was 20 μL. The outlet of the DAD was connected to a Dawn Heleos 8+ MALS/QELS system (Wyatt Technology Corporation, Santa Barbara, CA, United States). MALS and QELS data were analyzed with the ASTRA V software (Version 5.3.4.15, Wyatt Technology Corporation, Santa Barbara, CA, United States) and resulted in root mean square radius (rms) and molecular weight (both assessed by MALS) and hydrodynamic radius (assessed by QELS). For protein separation and quantitation, a Caliper LabChip GX II (PerkinElmer, Waltham, MA, United States) high-throughput capillary gel electrophoresis (HT-CGE) device was employed. An HT Protein Express LabChip and the corresponding HT Protein Express Reagent Kit were used and results analyzed with LabChip GX software (Version 4.2.1745.0, PerkinElmer, Waltham, MA, United States). Analyses were performed using the HT Protein Express 200 assay in reduced mode using dithiothreitol (DTT, Amresco, Solon, OH, United States) according to the assay standard operation procedure provided by the manufacturer. For data analysis, all peaks of 21.5 ± 1 kDa were regarded as HBcAg monomers, which is the form in which HBcAg is present after sample preparation. The range derived from experiments with pure HBcAg. For SDS PAGE, LDS sample buffer, MES running buffer, and NuPage 4–12% BisTris Protein Gels were used and run on a PowerEase 500 Power Supply (all Invitrogen, Carlsbad, CA, United States) in reduced mode with 50 mM DTT in the sample solution according to the manufacturer’s manual with minor adaptations. The gel was stained with a Coomassie blue solution. CFF re-dissolution samples of fractions with maximum concentration were analyzed by transmission electron microscopy (TEM) on a Fecnei Titan^3^ 80–300 microscope (FEI company, Hillsboro, OR, United States). Samples were adjusted to 0.5–1 g/L with ultrapure water and filtered with a 0.2 μm syringe filter. Sample preparation and image analysis were conducted similarly to previous studies with chimeric HBcAg VLPs (Rüdt et al., 2019). Hydrophilization and staining solutions were 1% (w/v) alcian blue 8GX (Alfa Aesar, Ward Hill, MA, United States) in 1% acetic acid solution and 2% ammonium molybdate(VI) (Acros Organics, Geel, Belgium) solution (pH 6.25, adjusted with NaOH), respectively.

### Calculation of Yield, Purity, and Productivity Measures

The yield *Y* of a process was calculated by

(1) $$\begin{array}{c} Y=\frac{\sum_{\text{i}=\text{start}}^{\text{end}}m_{{\text{F}}_{\text{i}}}}{m_{\text{lysate}}}, \end{array}$$

where *m*_*lysate*_ is the mass of HBcAg, calculated from the processed lysate volume and HBcAg concentration as determined by HT-CGE, and *m*_*F_i*_ is the mass of HBcAg in re-dissolution fraction F as determined by SEC, where fractions were considered from fraction F_start_ to F_end_. HT-CGE purity was determined by the ratio of HBcAg concentration to total protein concentration in HT-CGE samples. SEC purity was calculated by the ratio of HBcAg peaks to total peak area at 280 nm (for details on peak identification, the reader is referred to Supplementary Information S3). A260/A280 was calculated by dividing the cumulated peak areas at 260 nm by the cumulated peak areas at 280 nm. Absolute spatial productivity *P* was calculated by

(2) $$\begin{array}{c} P=\frac{m_{\text{HBcAg},\text{recovered}}}{t_{\text{process}}}, \end{array}$$

where *m*_*HBcAg,recovered*_ is the accumulated mass of pooled fractions and *t*_*process*_ the time to complete the process starting with precipitated material through to recovery of the product. Relative spatial productivity was derived by the ratio of absolute productivities to the absolute productivity of the *Reference* process.

## Results

### Precipitation

**FIGURE 4:**
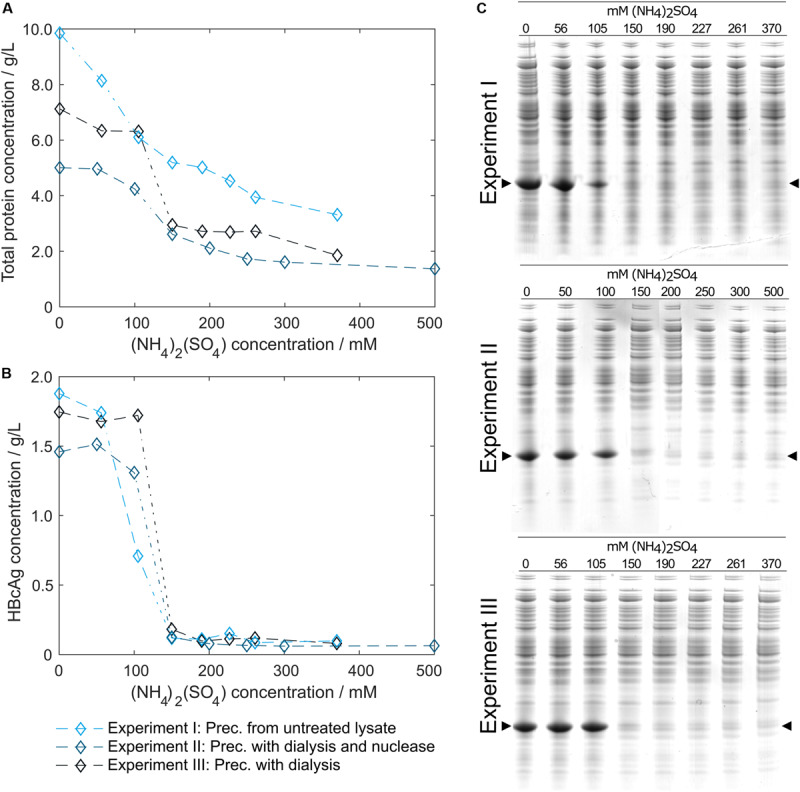
Total protein and hepatitis B virus core antigen (HBcAg) concentration in the supernatant after precipitation depending on ammonium sulfate [(NH_4_)_2_SO_4_] concentration. Total protein concentration by reducing high-throughput capillary gel electrophoresis (HT-CGE) is shown in **(A)**, HBcAg concentration by HT-CGE in **(B)**. Experiments I-III represent precipitation (Prec.) from (I) lysate (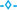), (II) lysate with added Benzonase dialyzed against digestion buffer overnight (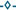), and (III) lysate dialyzed against digestion buffer overnight without addition of Benzonase (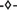). Experiments I-III are also shown as reducing SDS PAGE scans **(C)**, where lanes 1–8 show (NH_4_)_2_SO_4_ concentrations. The HBcAg band is indicated by arrows.

In pre-experiments, 150 mM (NH_4_)_2_SO_4_ was determined as optimal concentration for all process variants, where most of the product is found in the precipitate. Figure 4 shows HT-CGE and SDS PAGE data of the clarified supernatant of small-scale precipitation experiments from (I) lysate, (II) lysate with added Benzonase dialyzed against digestion buffer overnight, and (III) lysate dialyzed against digestion buffer over night without addition of Benzonase. The total protein concentration in the supernatant (Figure 4A) was higher for almost all (NH_4_)_2_SO_4_ concentrations for precipitation from untreated lysate than for dialyzed samples, as had been expected due to depletion of molecules during dialysis. HBcAg concentrations in all three experiments (Figure 4B) were comparable, except for the region between 100 and 150 mM (NH_4_)_2_SO_4_, where supernatant HBcAg concentrations during precipitation from non-dialyzed lysate dropped significantly at 100 mM (NH_4_)_2_SO_4_, while the dialyzed samples remained at comparably constant HBcAg concentrations from 0 to 100 mM (NH_4_)_2_SO_4_. SDS PAGE analysis (Figure 4C) showed similar results based on band intensities.

**FIGURE 5:**
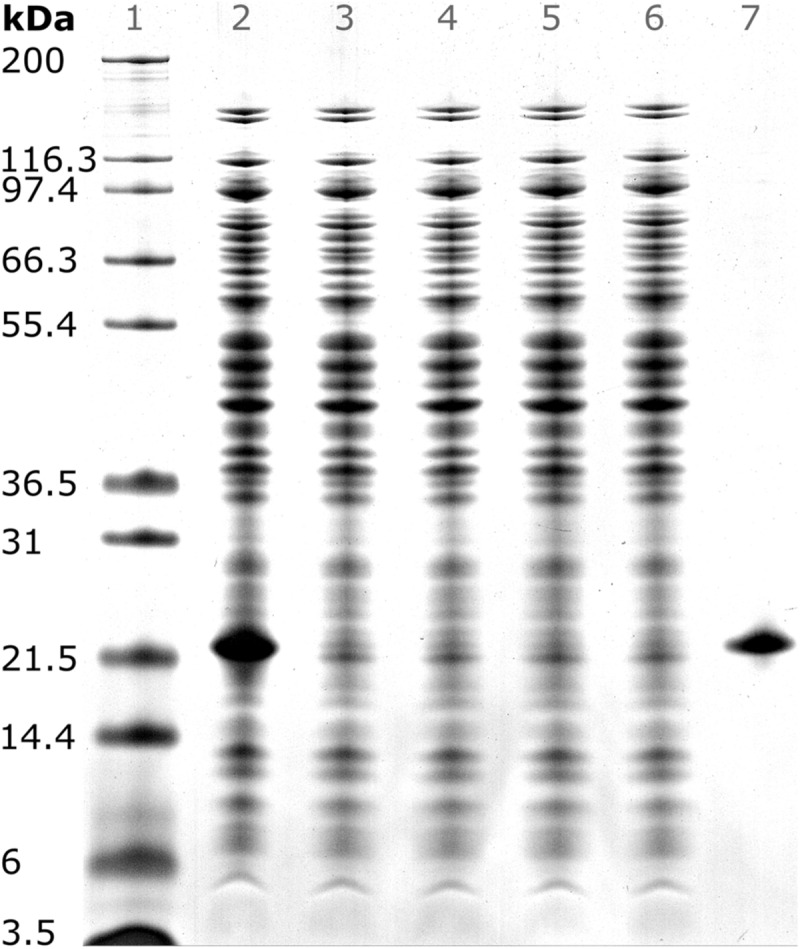
SDS PAGE scan of (1) Invitrogen Mark 12 Unstained Standard, (2) hepatitis B virus core antigen (HBcAg)-containing *E. coli* lysate, (3–6) supernatant of precipitation experiments with 150 mM ammonium sulfate directly, 10, 20, and 30 min after ammonium sulfate addition, and (7) pure chimeric HBcAg sample. Molecular weights of the proteins contained in the standard are shown on the left.

To validate that precipitation incubation time is sufficient at larger scale, HBcAg concentration in the supernatant was investigated in 10 min intervals at the previously determined 150 mM (NH_4_)_2_SO_4_. Precipitation of HBcAg was already completed directly after addition of (NH_4_)_2_SO_4_, judging visually based on SDS PAGE scans (Figure 5). It has to be noted that to the first sampling time 2–3 min have to be added, accounting for drawing of samples, transferring the samples into reaction tubes, and centrifugation of the samples. Interestingly, during titration of the untreated lysate with (NH_4_)_2_SO_4_, we observed a rapid increase in turbidity when a concentration of 100 mM (NH_4_)_2_SO_4_ was exceeded. Nevertheless, 150 mM (NH_4_)_2_SO_4_ and a precipitation duration of 30 min were chosen to include a safety margin, which was successful in all processes.

### Centrifugation-Based Reference Process

**FIGURE 6:**
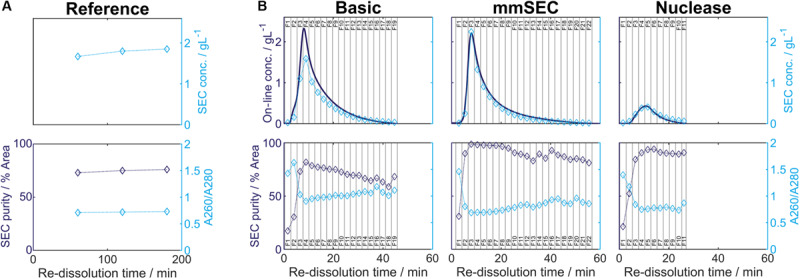
Re-dissolution protein concentration (conc.) and purity. Each figure column represents a re-dissolution process variant: **(A)** *Reference* and **(B)** *Basic*, *mmSEC* and *Nuclease*. In subfigure **(A)**, the *Reference* process concentration and purity data is shown based on off-line analysis of the supernatant after centrifugation. Top row: Off-line concentrations (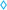) were derived from size-exclusion chromatography (SEC) peak areas of hepatitis B virus core antigen (HBcAg) species (Supplementary Information S3). Bottom row: SEC purity (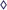) is defined as percentage of HBcAg peak area at 280 nm with respect to the area of all SEC peaks at 280 nm. A260/A280 (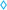) is defined as quotient of the cumulated SEC peak areas at 260 and 280 nm, respectively. Dotted lines are added to guide the eye. In subfigure **(B)**, on-line monitoring of the permeate concentration and off-line analysis of the corresponding permeate fractions (F, indicated by vertical lines) during CFF are shown. The metrics of subfigure **(A)** are shown in subfigure **(B)** using the same symbols. Additional to these metrics, protein concentrations (–) are shown. Protein concentrations are based on absorbance at 280 nm assuming the chimeric HBcAg extinction coefficient.

After precipitation, solid-liquid separation aims at separating the contaminant solutes and precipitation buffer from the precipitated product. A wash step increases the efficiency of contaminant removal. The *Reference* process was based on centrifugal solid-liquid-separation for precipitate recovery, wash, and re-dissolution. HBcAg concentration of re-dissolution supernatant increased over the first 3 h and was 1.67, 1.80, and 1.85 g/L, respectively (Figure 6A). Table 1 shows the re-dissolution concentration and purity measures after 3 h, where SEC purity was 76%, HT-CGE purity was 83%, and A260/280 was 0.87. After precipitation, which was conducted identically for all CFF processes and the *Reference* process, the *Reference* process was completed in 4.5 h. Time-specific productivities of all processes were calculated based on mg HBcAg per hour relative to the *Reference* process productivity. Therefore, the relative productivity of the *Reference* process is 100%, as shown in Table 1. Assuming a similar area foot print of the unit operations, a spatial component of the productivity was neglected.

**TABLE 1:** Summary of re-dissolution process data for centrifugation (*Reference*) and cross-flow filtration (*Basic*, *mmSEC*, *Nuclease*) processes. Process data above the thin horizontal border are calculated based on a pool of all fractions. Results below this border are based on a fraction pool that aimed for a product concentration of at least 1 g/L and a maximum yield. This was not possible for the *Nuclease* process. Values are calculated using total hepatitis B virus core antigen concentrations except A260/A280, which is based on all species in the size-exclusion chromatography (SEC) chromatogram (Supplementary Information Figure S3.1). Best results of each table column are printed in bold.

	**Mass^**†**^ (mg)**	**Yield^**‡**^ (%)**	**Conc.^**†**^ (gL^–1^)**	**SEC purity^**†**^ (% Area)**	**A260/A280^**†**^ (-)**	**HT-CGE purity (%)**	**Relative productivity^**†**^ (%)**
*Reference*	30.73	72	**1.85**	76	0.87	83	100
*Basic*^CFF,§^	36.26	82	0.38	73	1.02	96	264
*mmSEC*^CFF,§^	**37.82**	**86**	0.34	96	0.73	96	239
*Nuclease*^CFF,§^	9.72	22	0.18	86	0.82	**98**	8
*Basic*^CFF,¶^	25.19	57	1.01	78	0.96	95	248
*mmSEC*^CFF,||^	30.01	68	1.00	**98**	**0.70**	96	**269**

*CFF, cross-flow filtration process, ^†^assessed by SEC, ^‡^for definition see section “Materials and Methods” Eq. 1, ^§^pool of all fractions, ^¶^pool of fractions F3–F7, ^||^pool of fractions F3–F8. Process data for pools were calculated by accumulating fraction process data. A260/A280: absorbance ratio of the sample at 260 to 280 nm; Conc., concentration; HT-CGE, high-throughput capillary gel electrophoresis; SEC, size-exclusion chromatography.*

### CFF-Based Wash and Re-dissolution Processes – On-Line Monitoring and Off-Line Analysis

While in the centrifugation-based *Reference* process, wash, re-dissolution, and product recovery steps have to be performed individually (Figure 2, *Reference*), the CFF set-up allows for process step integration. Diafiltration with a wash buffer retains the product while depleting solutes continuously. Diafiltration into a re-dissolution buffer replaces the wash/precipitation buffer and re-dissolves the product, which is then able to pass the 0.2 μm membrane. This additionally ensures that larger particles, such as insoluble precipitate, are removed by retention. The developed set-up facilitates fractionation of the permeate stream enabling individual analysis of the fractions (Figure 3).

In the presented CFF processes, the wash step was stopped when the initially saturated on-line UV absorbance in the permeate fell below 4 mAU (for visualization of this process see Supplementary Information S4). Product loss during the wash step was determined by HT-CGE. HBcAg concentrations in wash fractions were 0.02–0.03 g/L. The additional wash step prior to precipitation of the *Nuclease* process resulted in less than 0.1 mg HBcAg loss (analyzed by SEC). After precipitation and wash, re-dissolution of the product was initiated by switching DF buffer lines from wash buffer to re-dissolution buffer. Figure 6B depicts on-line and off-line process data over time for the re-dissolution step in the three CFF process variants. Upon DF into re-dissolution buffer, on-line permeate concentrations for all process variants increased to a maximum after a lag phase of nearly 2 min and subsequently decreased exponentially. The process was stopped as soon as the on-line absorbance dropped below 4 mAU (on-line concentration of 0.01 g/L). The final retentate was analyzed for unrecovered product by HT-CGE. It showed a negligible HBcAg mass of <0.5 mg for processes *Basic* and *mmSEC*, as opposed to 22.4 mg in the *Nuclease* process. The maximum on-line concentrations were 2.3, 2.2, and 0.4 g/L for processes *Basic*, *mmSEC*, and *Nuclease*, respectively. The curve shapes of the off-line HBcAg concentration are in good agreement with the on-line data. In all three CFF processes, SEC purities were the lowest in fraction F1 and constantly increased to the purity maximum which coincided with the concentration maximum. Maximum purities were 82, 99, and 94% for processes *Basic*, *mmSEC*, and *Nuclease*, respectively. The SEC A260/A280 coefficient showed a nearly inverse progression compared to SEC purity data.

### Comparison of Process Data

As seen from summarized process data (Table 1), processes *Basic* and *mmSEC* showed higher HT-CGE purities and VLP yields compared to the *Reference* process. SEC purity was comparable between the *Reference* and the *Basic* process, while it was highest for the *mmSEC* process. The *mmSEC* process also showed lowest A260/A280 with 0.73. The relative productivities of processes *Basic* and *mmSEC* were higher than the *Reference* and the *Nuclease* process with >239%. While processes *Basic* and *mmSEC* were superior with regard to aforementioned process data, their concentrations were lower with 0.34–0.38 g/L as compared to 1.85 g/L for the *Reference* process. To increase pool concentrations, higher concentrated fractions can be selected for pooling. Strategic pooling increased concentrations for processes *Basic* and *mmSEC* to 1 g/L while maintaining purity and productivity. However, the yield decreased to 57–68%. Overall, the *mmSEC* process showed highest recovered mass, yield, SEC purity, and lowest A260/A280, along with high productivity and HT-CGE purity, both for strategic pooling and pooling of all fractions.

The *Nuclease* process showed great product loss during re-dissolution, as mentioned above. It exhibited the lowest yield and relative productivity of 22 and 8%, respectively. Due to low concentrations, purity is not comparable to the other processes. For completeness, these values are plotted in Figure 6B and shown in Table 1. Compared to the other processes, the precipitation process following nuclease treatment started with altered solution conditions regarding NaCl, MgCl_2_, EDTA, and impurity concentrations. Five screening experiments were designed to investigate the influence of solution conditions during precipitation on re-dissolution efficiency. The recovery of HBcAg in the re-dissolution experiments was 82 ± 1%, indicating no significant difference in HBcAg recovery between the investigated experimental conditions.

### VLP Size Analysis

**FIGURE 7:**
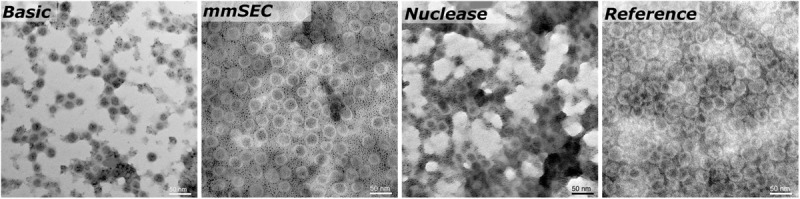
Transmission electron microscopy micrographs of re-dissolution peak samples of four processes: *Basic*, *mmSEC*, *Nuclease*, and the *Reference* centrifugation process. The magnification was 27000-fold.

SEC, coupled to DAD, MALS, and QELS, detected three peaks attributed to HBcAg (compare Supplementary Information S3 for peak identification). A main peak was identified with 15.3–15.5 nm rms radius and 16.4–17.7 nm hydrodynamic radius, corresponding to 79–84% of the HBcAg peak area in the CFF processes. In the *Reference* process, it was 65%. The two earlier-eluting peaks showed 24.4–25.2 nm and 30.4–32.0 nm radius, respectively. The molecular weights were 3.8–4.1 MDa, 7.5–7.8 MDa, and 12.2–12.7 MDa for the three peaks in ascending order by radius. Figure 7 shows TEM micrographs of the processes *Basic*, *mmSEC*, *Nuclease*, and the *Reference* process. Graphical analysis resulted in average radii of 13.4 ± 1.2, 14.6 ± 1.5, 13.6 ± 1.2, and 15.3 ± 1.8 nm, respectively, not showing distinct species as observed in SEC. While samples from processes *mmSEC* and *Reference* showed a spatially equal distribution of VLPs, *Basic* and *Nuclease* samples appeared clustered.

## Discussion

### Interpretation of Analytical Methods

In this study, SEC and HT-CGE have been applied to determine concentrations and to identify the quantified species. It is therefore important to discuss the meaning of the analytical data as determined for the presented processes. HT-CGE has been employed as, compared to SDS PAGE, a high-throughput compatible and quantitative size-dependent concentration analytical technique. HT-CGE purity informs about the relative HBcAg fraction of the total protein content, i.e., HBcAg protein purity. SEC is applied to assess particle size and molecular weight, HBcAg and contaminant concentrations, and additionally provides spectral data of the sample.

The ratio of the absorbance at 260 nm to the absorbance at 280 nm (A260/A280) is characteristic for the ratio of nucleic acid to protein concentration, whereby higher A260/A280 values indicate a larger fraction of nucleic acids (Wilfinger et al., 1997). SEC purity describes purity based on all species absorbing at 280 nm, such as proteins and nucleic acids.

**FIGURE 8:**
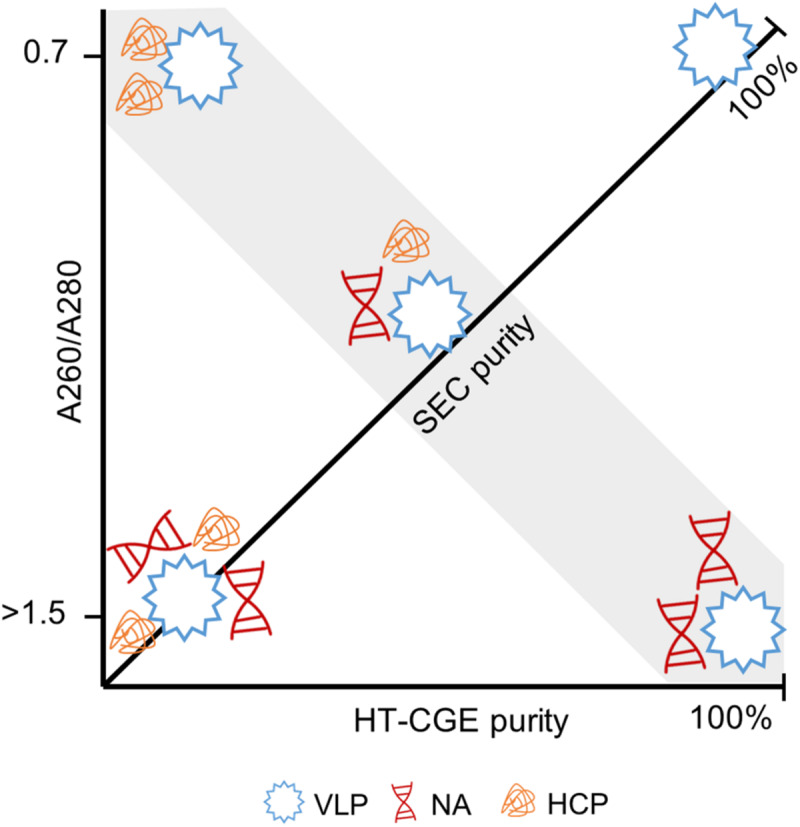
Illustration of the interdependence of derived purity measures. Virus-like particles (VLPs) with different degree of contamination by host cell proteins (HCPs) and nucleic acids (NAs) are shown. Size-exclusion chromatography (SEC) provides the A260/A280 (ordinate) and SEC purity (diagonal axis). A high-throughput capillary gel electrophoresis (HT-CGE) protein assay provides the HT-CGE purity (abscissa). The gray highlighted area is characterized by identical SEC purity, while HT-CGE purity and/or A260/A280 describe the composition of the contaminants. A pure hepatitis B virus core antigen VLP sample is characterized by 100% SEC purity, 100% HT-CGE purity and an A260/A280 of ∼0.7.

The combination of these two purity measures together with the A260/A280 are thus seen to be powerful to describe a sample. Figure 8 illustrates the connection between these measures. For example, samples with high HT-CGE purity but lower SEC purity therefore probably also show increased A260/A280 values, indicating nucleic acid contamination. It is important to note that SEC measurements are more accurate than HT-CGE measurements for concentration determination. This being said, SEC could only be applied to rather clean, non-turbid samples (see also Supplementary Information S5.1). Therefore, SEC rather was applied to assess concentrations during re-dissolution while lysate and precipitation/wash samples were assessed by HT-CGE. Yields were calculated based on lysate HBcAg concentrations and re-dissolution sample concentrations and are therefore based on both HT-CGE and SEC measurements. Discussion on comparability of yields can be found in Supplementary Information S5.2.

Off-line SEC and HT-CGE analysis indicated that mainly HBcAg species pass through the membrane upon re-dissolution. It was therefore reasonable to convert the on-line UV absorbance into an on-line HBcAg concentration value, applying the HBcAg extinction coefficient. The good agreement between on-line and off-line concentration profiles underlines the usefulness of this approach. However, the *mmSEC* process set-up included an additional purification step between the UV flow cell and the fraction collector, making off-line samples purer than the on-line measured permeate stream.

The MALS detector coupled to the SEC system provides an estimate of molecular weight. HBcAg capsids naturally occur as 180-mer with icosahedral symmetry *T* = 3 and as 240-mer with symmetry *T* = 4 (Wynne et al., 1999). As SEC is incapable of separating different capsid symmetries, the molecular weight measured is the average weight of *T* = 3 and *T* = 4 capsid species. The theoretical molecular weight for a chimeric *T* = 4 capsid is 4.8 MDa and a *T* = 3 capsid is 3.6 MDa. The SEC-MALS-derived molecular weights of the latest-eluting HBcAg peak were between 3.8 and 4.1 MDa, representing 18/82% and 43/57% mixture of *T* = 3/*T* = 4 capsids, respectively. *In vitro*, HBcAg VLPs are predominantly *T* = 4, but can shift toward higher percentage of *T* = 3 symmetry capsids upon VLP modification (Zlotnick et al., 1996; Böttcher et al., 1997; Rybka et al., 2019). As an orthogonal method, TEM imaging confirmed the presence of approximately 30 nm sized nearly spherical particles. TEM image-based size measurements did not result in significant differences between the VLP sizes in samples of the different processes. Due to graphical sizing inaccuracies, TEM was unable to resolve different HBcAg species as observed with SEC. These three differently sized HBcAg species, of which the smallest corresponds to the typical size of an HBcAg VLP, were observed in all CFF processes and the *Reference* process. Interestingly, the VLP fraction of these three peaks was similar in all the CFF processes but higher than in the reference process. It would be interesting to analyze these species separately in the following process steps, such as disassembly, which is, however, out of the scope of this study.

### Precipitation of Chimeric HBcAg VLPs

Precipitation of complex mixtures involves interactions that are only partly understood (Przybycien, 1998). This has also recently been pointed out in a study on PEG-induced precipitation of mAbs (Großhans et al., 2019). Although differences were small in our study, variations of HBcAg concentrations were observed especially at 100 mM (NH_4_)_2_SO_4_, where supernatant concentrations after precipitation from untreated lysate were lowest. This is in accordance with previously reported results on mAb precipitation from complex mixtures in the study mentioned above, where precipitation from a complex mixture led to higher precipitation propensity of product molecules (Großhans et al., 2019). This rapid decrease in HBcAg solubility at 100 mM concurs with the observed rapid turbidity increase at 100 mM (NH_4_)_2_SO_4_ at a larger scale during the CFF and centrifugation processes. Experiments on precipitation incubation time revealed that the investigated HBcAg VLPs precipitate almost immediately, which is fast compared to incubation times of 15 min – 4 h for different VLPs and precipitants stated in literature (Schagen et al., 2000; Tsoka et al., 2000; Koho et al., 2012).

### Product Loss in the Nuclease Process

The *Nuclease* process showed significantly lower concentrations of recovered HBcAg, making it difficult to compare this process variant to the other processes. Due to its low relative productivity and comparably complicated process route, it is not competitive with the *Reference* process and the other CFF processes *Basic* and *mmSEC*. The low yield observed in this process is mainly due to incomplete re-dissolution, with 22.4 mg of HBcAg in the final retentate. In order to reveal the effect of different solution conditions during the precipitation step, this was investigated in small-scale re-dissolution experiments. However, no significant differences could be identified when investigating the influence of NaCl, EDTA, MgCl_2_, and contaminants with regard to this problem. Further reasons could be the additional wash step by DF on a membrane of different material or overnight incubation at RT, resulting in irreversible precipitation. Apart from low yields, its low relative productivity derives from the 16 h Benzonase incubation, yet only increases to 42% if an incubation time of 1 h at optimized digestion conditions would be considered. From a scientific standpoint, it would be interesting to identify which factors contributed to the low re-dissolution yields, whereas from a technical standpoint this process route cannot be justified.

### Benefits of Process Transfer to a CFF Unit

The main advantage in implementing CFF for precipitation/re-dissolution lies in the combination of product recovery by membrane retention with the capability of exchanging the product-containing buffer in a single process step. During CFF wash steps, impurities smaller than 0.2 μm are expected to be washed out with the permeate. Impurity depletion was observed in all processes indicated by the decrease of on-line UV absorbance. HBcAg VLPs are expected to be retained by the membrane due to the size of their precipitate, as was seen for mAb precipitate in previous studies (Kuczewski et al., 2011; Hammerschmidt et al., 2016). Although HT-CGE results point at minor product loss during wash, it is important to note, that all proteins of 19.5–21.5 kDa were assigned to HBcAg in our analysis due to sizing inaccuracies. Therefore, product loss is expected to be lower than reported. The wash process step was comparable for processes *Basic* and *mmSEC*. Higher protein purities in the CFF processes are probably due to a more efficient wash as compared to the centrifugation-based *Reference* process, whereby interstitial pellet liquid cannot be removed. However, in the *Basic* process, SEC purity was slightly lower and A260/A280 higher than in the *Reference* process. This indicates that the main impurity in the *Basic* process are nucleic acids. This is in accordance with previous unpublished results of CFF-based processes from our group. It may be suggested that DNA interacts with the VLPs in the kosmotropic environment during precipitation and wash which hampers its depletion during the wash step.

As opposed to re-dissolution of the compact pellet in the *Reference* process, re-dissolution from a turbid solution in CFF-based processes was expected to improve process performance. This was for example observed by the increased yields of processes *Basic* and *mmSEC* compared to the *Reference* process. Product loss in the *Reference* process can be attributed to unrecoverable interstitial pellet liquid and high precipitate compaction (Hammerschmidt et al., 2016), which leads to slower and incomplete re-dissolution. This is in agreement with comparably slow re-dissolution in the *Reference* process. As a result, CFF processes *Basic* and *mmSEC* showed strongly enhanced relative productivities. Additionally, CFF process durations are reduced by minimizing manual handling compared to the *Reference* process. The *mmSEC* process showed superior SEC purity compared to all other processes. As discussed above, the main contaminant in the *Basic* process are nucleic acids. These were efficiently depleted in the *mmSEC* process, leading to excellent purity, while maintaining the increased yield of the *Basic* compared to the *Reference* process, underpinning the usefulness of the mmSEC column in the permeate line (Figure 3).

In summary, process transfer to the CFF set-up led to improved yields, accelerated re-dissolution kinetics, and process intensification by integrating multiple process steps into one unit operation. Compared to literature VLP processes showing a 31–76% recovery (Zhao et al., 2015; Carvalho et al., 2019), up to 95% protein purity (Wetzel et al., 2018), and a 78% nucleic acid reduction (Carvalho et al., 2019), the process data of the *mmSEC* process are comparable or superior while applying only a single unit operation after lysate clarification. The main drawback of the CFF-based processes were lower product concentrations as compared to the *Reference* process. The exponential permeate concentration decrease observed for all re-dissolution processes, as expected for non-retained species in DF (Kurnik et al., 1995), results in decreased concentrations when aiming for a maximized process step yield. Although the re-dissolution concentration profile cannot be improved from a technical point of view, this effect can be ameliorated by strategic pooling. This was exemplified by creating 1 g/L pools, which resulted in improved purity and 18–25% yield decrease. Alternatively, collection of all fractions followed by a concentration process via UF could maximize both yield and concentration. Another interesting option would be loading the permeate onto an anion exchange column or membrane as a polishing step to bind VLPs, deplete (NH_4_)_2_SO_4_, and achieve further purification from other contaminants while obtaining concentrated VLPs in the elution step. While it seems reasonable to dissolve the precipitated product by dilution to avoid DF-associated concentration decrease, DF shows several advantages. Considering 0% retention, 40% of (NH_4_)_2_SO_4_ is theoretically found in fractions 1–2, which could be discarded due to low VLP concentrations. On the contrary, all (NH_4_)_2_SO_4_ remains in the product solution for re-dissolution by dilution as used in several concepts for mAb capture processes (Kuczewski et al., 2011; Hammerschmidt et al., 2016; Li et al., 2019). This drawback may be circumvented by employing dead-end filtration to drain precipitate before re-dissolution (Chen et al., 2016; Liu et al., 2019; Lohmann and Strube, 2020). This approach was not considered in this study to avoid unknown effects of draining, precipitate compaction on the membrane, and uncontrolled concentration increase on product stability and yield. DF allows for highly efficient (NH_4_)_2_SO_4_ removal in the retentate enabling maximum re-dissolution and therefore yield. Conversely, comparable levels of (NH_4_)_2_SO_4_ can only be reached by dilution to very large volumes. Especially if a UF step is established after re-dissolution, a simple DF step after concentration can remove residual (NH_4_)_2_SO_4_ efficiently.

To the best of our knowledge, this is the first study to present a fully integrated CFF system-based precipitation, wash and re-dissolution set-up for VLP capture and purification that includes DF-based re-dissolution. The presented approach showed exceptionally good performance with regard to yield, purity, and productivity while being based on a simple lab-scale set-up with basic commercial devices. As a filtration-based process, it exhibits good scalability and the possibility of disposable manufacturing (van Reis and Zydney, 2007). For vaccines, especially cancer vaccines, which are envisaged to be produced as personalized medicine (Buonaguro et al., 2013; Rammensee and Singh-Jasuja, 2013; Castiblanco and Anaya, 2015), this highly efficient, easy-to-control, and scalable process could enable distributed manufacturing of personalized protein nanoparticle-based therapeutics.

### Considerations for Method Transfer

From a technical point of view, CFF process control of the presented method can be achieved by maintaining a constant transmembrane pressure (TMP) or permeate flow rate. In case of TMP-based control, low TMP values are required to obtain the target permeate flow rate due to the large membrane pore size of 0.2 μm. During wash and re-dissolution in processes *Basic* and *mmSEC*, the TMP was in the range of 0.01 bar to 0.02 bar. Therefore, a careful adjustment of the TMP is recommended to avoid exceeding the maximum flow rate of the mmSEC column. Nevertheless, a constant flow rate is advantageous for fractionation and mmSEC separation.

The prerequisites for the successful application of this process to the purification of other VLPs are the ability (I) to precipitate the target product, (II) to retain the majority of impurities in solution, (III) to re-dissolve the product, and (IV) to avoid electrostatic or hydrophobic interaction between product and impurities or matrices, such as the membrane material. These prerequisites are probably fulfilled – to varying degrees – for most non-enveloped VLPs.

Precipitation of the target product might require adaption of the precipitant concentration or agent for different VLPs. From unpublished results of our group, we learned that the precipitation of other chimeric HBcAg VLPs required ammonium sulfate concentrations of 0.1 M to 1 M. Their large size compared to the typical contaminants facilitates the precipitation of VLPs while retaining most impurities in solution. The application of this process to smaller product molecules (such as capsomers) could also be feasible, if a suitable precipitation method is developed, which retains impurities in solution. Product re-dissolution and hydrophobic or electrostatic interactions are influenced by the solution conditions, which might need to be optimized, presumably with a focus on the optimum solution pH.

Compared to the here investigated non-enveloped VLPs, enveloped VLPs might pose a challenge due to their lower stability (Dai et al., 2018). VLPs derived from other hosts such as yeast or plants require changes in the lysis procedure and bring along a different impurity profile than *E. coli.* This said, the separation in the presented process is largely based on the size difference between product and impurities, which should be comparable for other hosts. Extracellularly produced VLPs could benefit from the higher purity of the starting material and therefore potentially result in yet higher purities using this process. Conclusively, the transfer of this method to the purification of other VLPs probably requires few adaptations, mainly regarding the development of optimal solution conditions for VLP precipitation and re-dissolution in small scale.

## Conclusion and Outlook

In this study, we have developed a set-up for integrated capture and purification of VLPs within a CFF unit. Clarified lysate was precipitated, washed, and re-dissolved. Three CFF process variants were investigated and characterized for yield, purity, and relative productivity and were compared to a centrifugation-based *Reference* process. Process transfer of the *Reference* process to the CFF unit led to increased purities, probably attributed to a more efficient wash step. The *mmSEC* process, integrating an additional purification step by an mmSEC column in the permeate line, was superior to all tested variants and the *Reference* process resulting in the highest purity and productivity. As one single unit operation, it compares favorably to entire DSP processes found in the literature and shows great potential for disposable and scalable manufacturing. Another key advantage of CFF processes is the possibility to fractionate the VLP-containing permeate, allowing for efficient pooling with regard to the desired target process data and product analytical profile. In the future, this mainly size-based DSP step could be applied to other VLPs or similarly sized therapeutics with only minor adaptations, laying the foundation for a platform process for protein nanoparticles.

## Data Availability Statement

The raw data supporting the conclusions of this article will be made available by the authors, without undue reservation, to any qualified researcher.

## Author Contributions

JH initiated and supervised the work. NB performed HT-CGE analysis of the samples. AD aided in performing ammonium sulfate screening experiments and CFF processes. NH and PV evolved the concepts and set-up presented in this manuscript, performed the experimental work, analyzed and interpreted the data, and drafted the manuscript. NH, PV, NB, AD, and JH read and approved the final manuscript.

## Conflict of Interest

The authors declare that the research was conducted in the absence of any commercial or financial relationships that could be construed as a potential conflict of interest.

## Abbreviations

(NH_4_)_2_SO_4_: ammonium sulfate
CFF: cross-flow filtration
DAD: diode array detector
DF: diafiltration
DSP: downstream processing
DTT: dithiothreitol
*E. coli*: *Escherichia coli*
HBcAg: hepatitis B virus core antigen
HCP: host cell protein
HT-CGE: high-throughput capillary gel electrophoresis
mAb: monoclonal antibody
MALS: multi-angle light scattering
mmSEC: multimodal size-exclusion chromatography
P&ID: piping and instrumentation diagram
PEG: polyethylene glycol
QELS: quasi-elastic light scattering
RT: room temperature
SEC: size-exclusion chromatography
TEM: transmission electron microscopy
TMP: transmembrane pressure
UF: ultrafiltration
UV: ultraviolet
UV/Vis: ultraviolet and visible light
VLP: virus-like particle.
