# Supplementary material for: Integrated Process for Capture and Purification of Virus-Like Particles: Enhancing Process Performance by Cross-Flow Filtration
Source: Front Bioeng Biotechnol. 2020 May 25;8:489. doi: 10.3389/fbioe.2020.00489 (PMC7326125; doi:10.3389/fbioe.2020.00489)
Supplement: Supplementary file 1 [file Data_Sheet_1.PDF]

Supplementary Material

S1 Chimeric HBcAg expression and cell lysis

The chimeric HBcAg construct was modified with a foreign epitope in the major immunodominant region and C-terminally truncated as previously described (Klamp 2011, Schumacher 2015). HBcAg protein was recombinantly overexpressed in *E. coli* BL21 DE3 (New England Biolabs, Ipswitch, US-MA). Expression was induced using a TB-based auto-induction medium developed by BioNTech Protein Therapeutics GmbH. Cells were cultured at 180 rpm and 37 °C for 7 h in a MaxQ 6000 Shaker (Thermo Scientific, Marietta, US-OH) with 250 mL medium in 1 L baffled glass shake flasks (Schott AG, Mainz, DE) up to an OD_{600} of 6. Cells were harvested by centrifugation at 4 °C at 3220 rcf for 30 min in an Eppendorf Centrifuge 5810 R (Eppendorf, Hamburg, DE), washing the pellet with phosphate-buffered saline at pH 7.4, and centrifugation at 4 °C at 17387 rcf for 20 min. Pellets were generated from 500 mL of culture volume and frozen at -30 °C for storage. For lysis, the pellet was thawed and resuspended in 20 mL of lysis buffer. Ultrasonic disruption was performed with a Digital Sonifier 450 (Branson Ultrasonic Corporation, Danbury, US-CT) at 80% amplitude for 2×40 s with a 3 min break. During this procedure, the sample was cooled in a stirred ice bath. Cell debris were separated from the supernatant by centrifugation at 4 °C and 17387 rcf for 20 min and filtration through a glass fiber and 0.45 µm cellulose acetate syringe filter (both Sartorius Stedim Biotech GmbH, Göttingen, DE). The lysate was stored at -30 °C. Prior to precipitation and re-dissolution experiments and processes, lysate was thawed and filtered again through a 0.45 µm syringe filter.

S2 CFF set-up and temporal alignment

In pre-experiments, flow rates for the CFF-DF steps were tested. Constraints were the linear range of permeate flowrate over TMP and the maximum tolerable flow rate of the mmSEC column. Resulting flowrates were 30 and 2 mL/min for feed and permeate flow rate, respectively. The pump of the ÄKTA Start chromatography system was bypassed and the flow generated and controlled by the CFF unit's backpressure valve. Setting the set-point as surrogate flow rate in the chromatography system settings was necessary to enable data collection and fractionation. Flow meter, chromatography fraction, and UV absorbance data were temporally aligned and processed volumes as well as fraction volumes were retrospectively corrected by integration of flow rate over time. Before integration, flow rate data were smoothed using a moving mean with a window of 50 data points, corresponding to 3 s. Delay volumes of the chromatography system were automatically corrected. For the *mmSEC* process, the column was inserted after the fractionation valve to ensure UV absorbance monitoring during the wash procedure avoiding flow over the column. Contrary to the other processes, the wash step permeate had to be collected from the wash valve before the column. Fractions were collected manually based on the flow meter cumulative volume readings. During re-dissolution, a volume of 1.96 mL was needed for VLPs to pass the mmSEC column and was therefore manually added to the delay volume during alignment. Manual and automatic flow rate control in all processes resulted in maximum 3% deviation of the mean flow rate from the set-point and a coefficient of

variation smaller than 9%. Flow rate data of the first three minutes showed transient oscillation and were omitted in the calculations.

S3 SEC analysis

Samples were separated by size using analytical SEC. Three detectors were coupled to the UHPLC system, which were a DAD, MALS detector, and QELS detector. The DAD recorded spectra as well as single wavelengths, of which 260 nm and 280 nm were used for SEC purity and A260/A280 calculation. A typical UV chromatogram is shown in Figure S3.1.

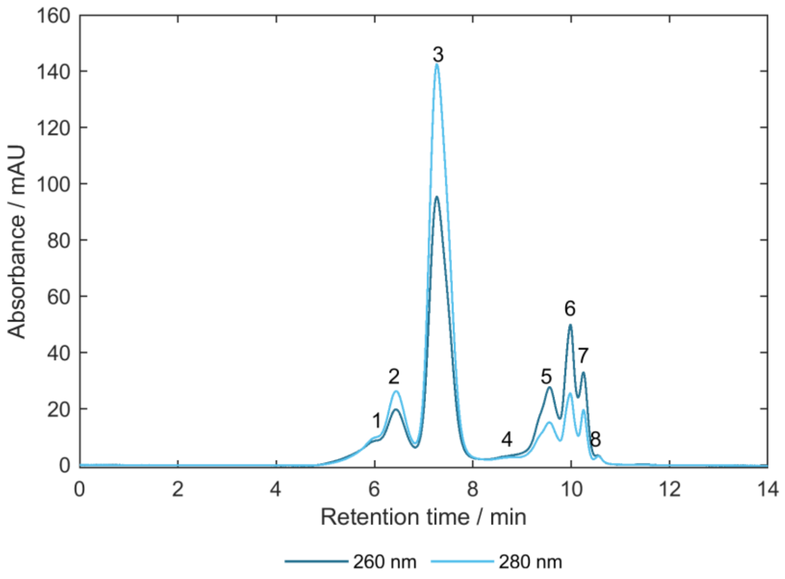

Figure S3.1: Size-exclusion chromatography chromatogram of the *Basic* process fraction F4 showing absorbance at 280 and 260 nm over retention time. Detected peaks are marked with numbers. Peaks 1-3 represent hepatitis B virus core antigen species; peaks 4-8 represent impurities.

Eight peaks were detected, whereby peaks 1-3, showing protein-typical A260/280 values of mostly <0.75, were attributed to HBcAg. This assumption was confirmed by HT-CGE analysis of samples that showed almost only peaks 1-3 (average 98% SEC purity, main text Table 1), such as samples of strategic pooling for process *mmSEC*. These samples exhibited one dominant peak in the HT-CGE electropherogram corresponding to monomeric HBcAg (average 96% HT-CGE purity, main text Table 1). During sample preparation for the protein HT-CGE assay, all proteins are denatured and reduced and therefore disassembled to monomers. It is therefore reasonable to assume that peaks 1-3 only differ in their quaternary structure while being based on HBcAg molecules. Peaks 4-8 showed higher absorbance at 260 nm and are therefore probably mainly nucleic acid species (Wilfinger, Mackey, and Chomczynski 1997). This scheme was observed for all CFF re-dissolution samples. For re-dissolution samples in the *Reference* process, peak 5 was dominated by protein contaminants, according to the UV spectral data

(A260/A280 < 1.0, data not shown), not seen in the CFF processes. This is in accordance with lower protein purities seen for the *Reference* process samples (main text Table 1).

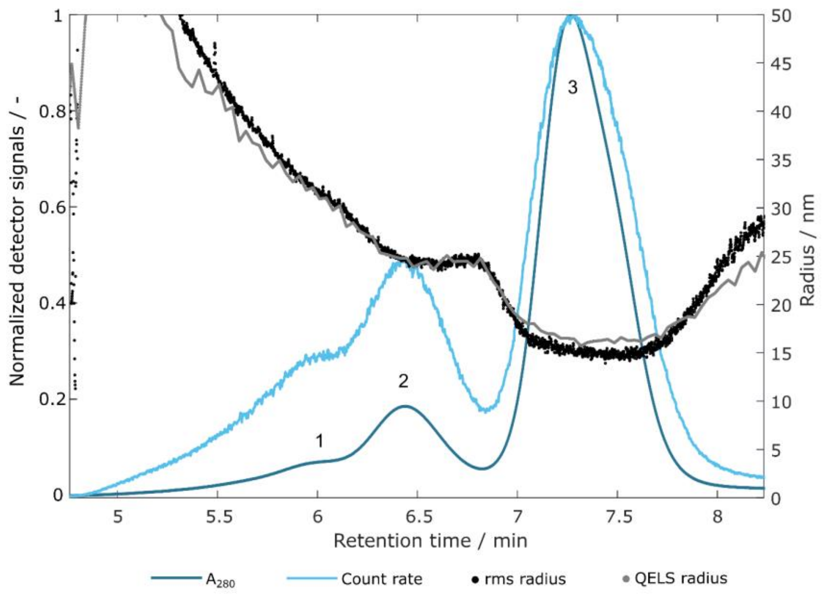

Figure S3.2: Absorbance at 280 nm and light scattering signals of a size-exclusion chromatography analysis of fraction F4 of the *Basic* process. Absorbance at 280 nm (A_{280}, —) and light scattering count rate (—) are normalized by their maximum value. Peaks 1, 2, and 3 are marked with numbers 1-3. Root mean square (rms, •) and quasi-elastic light scattering radius (QELS, •) are shown as absolute values.

Figure S3.2 shows an excerpt of the above shown SEC chromatogram with normalized signals of absorbance at 280 nm and count rate derived from the light scattering device focusing on peaks of HBcAg species. Root mean square (rms) radius and quasi-elastic light scattering (QELS) radius are shown which were calculated by a 1st degree Zimm model and by the manufacturers' QELS model, respectively. The size measurements were in good agreement and resulted in radii of 31-32 nm, 25 nm, and 15-16 nm for peaks 1, 2, and 3, respectively. Only peak 2 and 3 represent typical peak shapes and therefore likely represent a distinct species each, while peak 1 probably represents a broad range of aggregates of various sizes. The largest peak, peak 3, showed a radius typical for HBcAg VLPs (15-17 nm (Selzer and Zlotnick 2017)). Other chromatograms were almost identical, but tended to diverge more at very low sample concentrations due to a disadvantageous signal-to-noise ratio (data not shown).

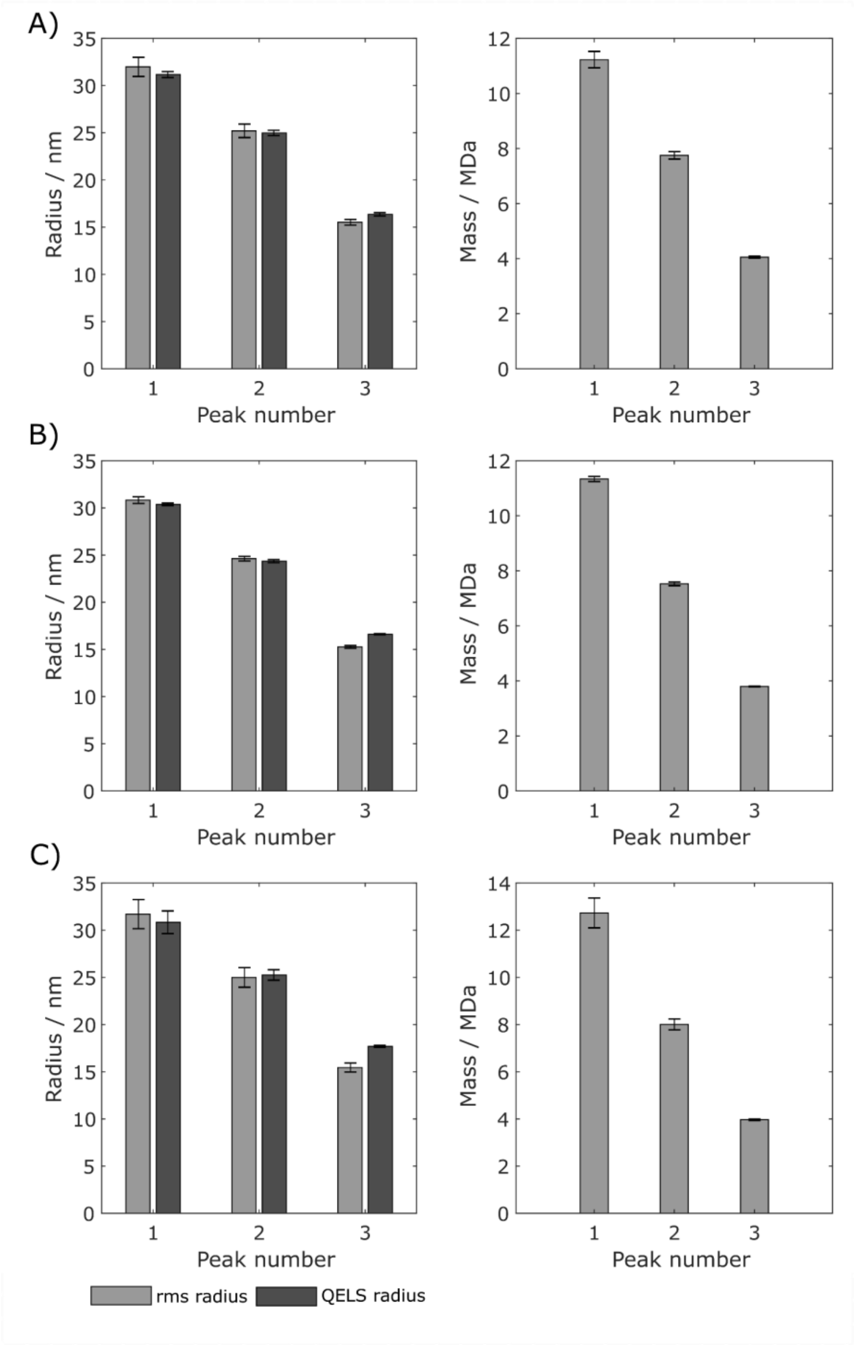

Figure S3.3: Size measurements of fractions with highest hepatitis B virus core antigen concentrations of the processes **(A)** *Basic*, **(B)** *mmSEC*, and **(C)** *Nuclease*. The left column shows root mean square radius (rms) and quasi-elastic light scattering (QELS) radius of peaks 1, 2, and 3, as indicated in Figure S3.2. The right column shows calculated mass of peak species 1-3. Error bars indicate standard deviations of cumulated measurement values within 0.15 min left and right of the SEC peak maximum from duplicate measurements.

Figure S3.3 shows size and mass of species behind peak 1-3 as indicated in Figure S3.2 for processes *Basic*, *mmSEC*, and *Nuclease* (Figure S3.3A-C). The difference between the processes was small but most notably between *Nuclease* and the other two processes. This is probably due to lower sample concentrations and therefore lower signal-to-noise ratio. As discussed above, peaks represent HBcAg species, which was concluded due to a low A260/280 ratio (~0.7), and high HT-CGE purity (≥96%), and typical protein UV spectra (not shown). QELS and rms radii are in good agreement. Only for peak 3 representing VLPs, QELS radius was slightly larger than rms radius, which is expected for spherical particles (Leszczyszyn 2012). In the following, peak radii are discussed indifferent of measurement type (rms or QELS) and processes. Peak 1 showed largest radius and weight with 30.4-32.0 nm and 11.2-12.7 MDa, respectively, and probably represents a broad size range of aggregates. The small range of the measured sizes for peak 1 is derived from the calculation method of peak data, which is based on a window of 0.15 min around the peak maximum as determined by SEC. Peak 2 was smaller with 24.4-25.2 nm and 7.5-8.0 MDa. Peak 3 was the smallest with 15.3-17.7 nm and 3.8-4.1 MDa. Its radius is consistent with HBcAg capsid size reported in literature.

Based on manual graphical size evaluation of TEM micrographs (main text Figure 7), it was not possible to identify distinct particle size species as seen with SEC (MALS/QELS), illustrating the limitation of this quantification method. The difference between the even distribution of VLPs in the *Reference* and *mmSEC* process and the observed VLP clusters in *Basic* and *Nuclease* process can most probably be caused by TEM grid preparation, sample adsorption, negative staining, and washing steps, rather than by differences in the samples. Existence of such clusters were not reflected by the results of SEC. SEC, as opposed to TEM measurements, reflects solution conditions and is therefore the preferred size analytical method.

S4 CFF wash and re-dissolution process data

Figure S4 depicts on-line concentrations for the processes *Basic*, *mmSEC*, and *Nuclease* showing both wash and re-dissolution process steps as a complement to Figure 6 in the main text. Initially, the signal was in saturation for processes *Basic* as well as *mmSEC* and decreased exponentially afterwards. The on-line concentration of the *Nuclease* process started below 1 g/L and also decreased exponentially. During wash, UV active impurities, such as proteins and nucleic acids, are depleted, leading to an elevated absorbance of the permeate at 280 nm which decreases over time. During the *Nuclease* process, the enzymatic digestion of nucleic acids and wash prior to precipitation leads to a lower initial contaminant level in the following wash step.

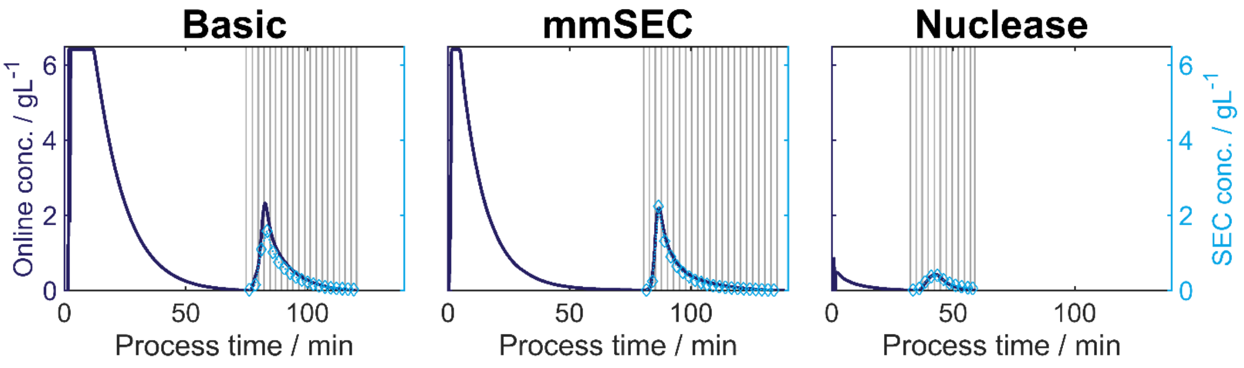

Figure S4: On-line monitoring of the permeate protein concentration (conc.) of wash and re-dissolution and off-line protein concentration of the re-dissolution fractions (indicated by vertical lines). Each column represents a process variant: Basic, mmSEC and Nuclease. Protein concentrations (—) are based on the absorbance at 280 nm, assuming the chimeric hepatitis B virus core antigen (HBcAg) extinction coefficient. Off-line concentrations (◊) were derived from size-exclusion chromatography (SEC) peak areas of HBcAg species (Figure S3.1).

S5: Analytical considerations

S5.1 Analysis of turbid samples

Turbidity prohibits analysis of the samples with SEC due to presence of precipitate that would block the column. During the *mmSEC* process, turbidity was observed in fractions F2 and F3, probably due to an erroneous priming of the mmSEC column with wash buffer, containing 150 mM $(NH_4)_2SO_4$. The $(NH_4)_2SO_4$ permeates slower through the column than VLPs, as it can penetrate the pores. VLP solution therefore leaves the column in a buffer with higher $(NH_4)_2SO_4$ concentration than before entering the column, thus leading to precipitation. This effect can be circumvented by priming the column in a non-$(NH_4)_2SO_4$-containing buffer. Upon dilution, the samples became clear and could be measured by SEC. Wash samples were measured by HT-CGE as, in particular for early samples, heavy precipitation was observed.

S5.2 Comparability of yields

Yields are calculated from re-dissolution and lysate HBcAg concentrations. Two separate methods have been employed to assess HBcAg concentration in the lysate and the re-dissolution samples, i.e. HT-CGE and SEC, respectively. SEC measurements exhibit much better reproducibility but could not be applied for lysate concentration measurements due to high impurity levels. Concentration determination by HT-CGE has a reproducibility of only 30% according to the manufacturer’s manual. Reasons for that include low-volume liquid handling of sample and buffers, interfering particles, and baseline determination. Yields relative to each other are well comparable due to highly reproducible SEC HBcAg concentration measurements of the re-dissolution samples. Additionally, HT-CGE assessed lysate HBcAg concentrations

were consistent between processes, which is owed to identical lysate preparation. However, absolute yield values are subject to variability related to HT-CGE reproducibility.

Supplementary Material References

Leszczyszyn, Oksana. 2012. "Hydrodynamic Radius Vs Radius of Gyration." Material Talks. 2012. http://www.materials-talks.com/blog/2012/11/15/size-matters-rh-versus-rg/.

Selzer, Lisa, and Adam Zlotnick. 2017. "Assembly and Release of Hepatitis B Virus," 1–18. https://doi.org/10.1101/cshperspect.a021394.

Wilfinger, William W, Karol Mackey, and Piotr Chomczynski. 1997. "Effect of PH and Ionic Strength on the Spectrophotometric Assessment of Nucleic Acid Purity." *BioTechniques* 22 (3): 474–81. https://doi.org/10.2144/97223st01.
